# Quantification and Analysis of Icebergs in a Tidewater Glacier Fjord Using an Object-Based Approach

**DOI:** 10.1371/journal.pone.0164444

**Published:** 2016-11-09

**Authors:** Robert W. McNabb, Jamie N. Womble, Anupma Prakash, Rudiger Gens, Christian E. Haselwimmer

**Affiliations:** 1 Geophysical Institute, University of Alaska Fairbanks, Fairbanks, Alaska, United States of America; 2 Glacier Bay Field Station, National Park Service, Juneau, Alaska, United States of America; Universita degli Studi di Milano-Bicocca, ITALY

## Abstract

Tidewater glaciers are glaciers that terminate in, and calve icebergs into, the ocean. In addition to the influence that tidewater glaciers have on physical and chemical oceanography, floating icebergs serve as habitat for marine animals such as harbor seals (*Phoca vitulina richardii*). The availability and spatial distribution of glacier ice in the fjords is likely a key environmental variable that influences the abundance and distribution of selected marine mammals; however, the amount of ice and the fine-scale characteristics of ice in fjords have not been systematically quantified. Given the predicted changes in glacier habitat, there is a need for the development of methods that could be broadly applied to quantify changes in available ice habitat in tidewater glacier fjords. We present a case study to describe a novel method that uses object-based image analysis (OBIA) to classify floating glacier ice in a tidewater glacier fjord from high-resolution aerial digital imagery. Our objectives were to (i) develop workflows and rule sets to classify high spatial resolution airborne imagery of floating glacier ice; (ii) quantify the amount and fine-scale characteristics of floating glacier ice; (iii) and develop processes for automating the object-based analysis of floating glacier ice for large number of images from a representative survey day during June 2007 in Johns Hopkins Inlet (JHI), a tidewater glacier fjord in Glacier Bay National Park, southeastern Alaska. On 18 June 2007, JHI was comprised of brash ice ($\bar{x}$ = 45.2%, SD = 41.5%), water ($\bar{x}$ = 52.7%, SD = 42.3%), and icebergs ($\bar{x}$ = 2.1%, SD = 1.4%). Average iceberg size per scene was 5.7 m^2^ (SD = 2.6 m^2^). We estimate the total area (± uncertainty) of iceberg habitat in the fjord to be 455,400 ± 123,000 m^2^. The method works well for classifying icebergs across scenes (classification accuracy of 75.6%); the largest classification errors occur in areas with densely-packed ice, low contrast between neighboring ice cover, or dark or sediment-covered ice, where icebergs may be misclassified as brash ice about 20% of the time. OBIA is a powerful image classification tool, and the method we present could be adapted and applied to other ice habitats, such as sea ice, to assess changes in ice characteristics and availability.

## Introduction

Tidewater glaciers, or glaciers that terminate in the ocean, are a prominent landscape feature in southeastern and south-central Alaska and play an important role in landscape and ecosystem processes. Changes in tidewater glaciers are cyclical in nature, with advance rates typically an order of magnitude slower and occurring over much longer time periods than retreats (e.g., [1,2]). Though these changes are generally in response to shifts in climate, the response of individual glaciers can vary greatly within individual climate regimes (e.g., [2]). Similar to terrestrially-terminating glaciers, most of the approximately 50 tidewater glaciers in Alaska have thinned and retreated over the last century [3–8], though some are currently relatively stable or even advancing [2]. Most models predict continual loss of glacier ice over the next century, though they currently do not account for frontal ablation (calving + submarine melt at the terminus), a major source of mass loss from tidewater glaciers [9]. As a result, the future availability of tidewater glacier ice for organisms that use the ice as habitat is largely unknown [10,11].

Through the fjord dynamics driven by melting at the calving front, as well as through calving itself, tidewater glaciers have a large impact on the fjord and marine environment [11]. Upwelling at the terminus provides nutrients, and fjords are consequently important habitats for fish, seabirds, and mammals, among other organisms [12,13]. Icebergs that emanate from tidewater glaciers serve as resting places for seabirds and provide important substrate for harbor seals (*Phoca vitulina richardii*) to rest, whelp, nurse young, molt, and avoid predators [13–15]. Because most tidewater glaciers in Alaska are thinning and retreating, there is concern regarding how changes or reductions in glacier ice may impact the organisms that rely on glacier ice as a habitat.

The availability of glacier ice is likely a key environmental variable that influences the abundance and distribution of harbor seals in tidewater glacier fjords in Alaska [16–18]. Previous studies of harbor seal ice habitat have estimated ice cover or indices of ice availability from oblique angles using shore- or vessel-based observers and photographs [17,19] or from videography collected during aerial surveys [18,20]. However, the amount of ice in the fjord and the fine-scale characteristics of ice including ice type, iceberg size, and iceberg angularity have not been systematically investigated. Given the predicted changes to tidewater glaciers, as well as other ice habitat, such as sea ice, there is a need to develop methods that could be broadly applied to quantify changes in available ice habitat in subpolar and polar regions, that provides important substrate for numerous species (e.g, [13, 21])

Object-based image analysis (OBIA) is a technique that incorporates spatial information from an image and breaks the image into smaller “image objects” that can be classified based on size, shape, or spectral characteristics (e.g., [22,23]). OBIA is a powerful image classification tool; however, it has been used only sparingly in the field of habitat classification (e.g., [24–27]). In contrast to traditional pixel-based classification approaches that use the individual spectral characteristics of each pixel, OBIA offers an enhanced ability to quantify morphological properties of habitat (e.g., size, shape, angularity). In addition, OBIA makes it feasible to quantify fine-scale features of habitats that are used by wildlife which are important in the context of elucidating relationships between wildlife and the habitats that they use. OBIA is also particularly beneficial for very high-resolution imagery where object features appear coherent and there are fewer mixed pixels [22,23].

Herein, we present a case study with glacier ice habitat in an Alaskan tidewater glacier fjord to describe a novel method that uses OBIA to quantify the amount and fine-scale characteristics of glacier ice from high resolution aerial imagery. Our primary objective was to develop a semi-automated method to estimate ice habitat from high spatial resolution airborne imagery collected of floating glacier ice in a tidewater glacier fjord. Specifically, we (i) developed workflows and rule sets to classify airborne imagery of glacier ice using object-based image analysis; (ii) quantified the amount and fine-scale characteristics of floating glacier ice in the fjord; (iii) developed processes for automating the object-based analysis of ice for a large number of digital images; and (iv) present results describing ice characteristics and accuracy assessments of classification from a representative survey day during June 2007. The described method could be applied to ice habitats, including sea ice, in subpolar and polar regions, that are used by other pagophilic species.

## Materials and Methods

### Ethics Statement

Aerial surveys were conducted under NOAA Fisheries Marine Mammal Protection Act permit #358-1787-01, and scientific permits issued by Glacier Bay National Park.

### Study area

Glacier Bay is an estuarine fjord in southeastern Alaska that constitutes part of Glacier Bay National Park (GLBA). GLBA is a marine protected area and is designated as an International Biosphere Reserve and World Heritage Site that encompasses over 2,400 km^2^ of marine waters [28].

**Fig 1:**
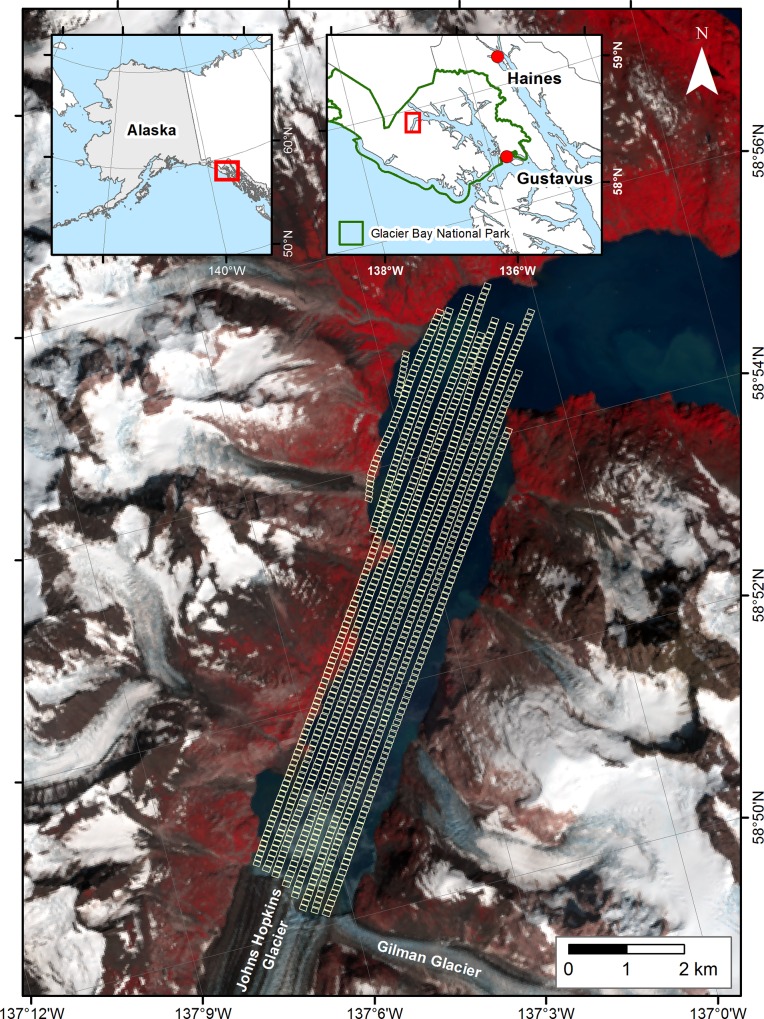
Map of Johns Hopkins Inlet study area. Johns Hopkins Inlet in Glacier Bay (top right inset), southeastern Alaska (top left inset). The standard false color composite image was created from a July 2013 Landsat 8 scene. Vegetated areas appear read and snow and ice appear in shades of white to grey. Individual image frames from the airborne survey along transect lines are plotted as small yellow squares.

Johns Hopkins Inlet (58.857856°N, 137.088841°W; Fig 1) is an expansive (~12 km long and ~2.5 km wide) tidewater glacier fjord in the upper West Arm of Glacier Bay. Johns Hopkins Inlet hosts the largest seasonal aggregation of harbor seals in Glacier Bay and is one of the primary glacier ice pupping and molting sites for harbor seals in southeastern Alaska [19,29]. Harbor seals rest on icebergs that have calved from two advancing tidewater glaciers: Johns Hopkins Glacier (250 km^2^; area estimates taken from the Randolph Glacier Inventory v3.3, [30]) and Gilman Glacier (25 km^2^), which extend from the Fairweather Range in the St. Elias Mountains to tidewater in Johns Hopkins Inlet.

After undergoing a retreat that began at the end of the 19th century, Johns Hopkins Glacier has advanced nearly 2 km since the mid-20th century, and is one of a few advancing tidewater glaciers in southeastern Alaska [2,4,31]. Since approximately 1988, Johns Hopkins and Gilman Glaciers have formed a single terminus. Several other small (< 10 km^2^) glaciers contribute seasonal freshwater input to the fjord, but no longer actively calve icebergs into the inlet [2]. Ice availability is typically highest in spring and early summer (May/June), when calving/frontal ablation tends to be the highest [32].

### Aerial surveys and image acquisition

The large expanse and remote location make it difficult to use traditional field-based survey methods to map the study site. High spatial resolution airborne images provide an alternate solution for mapping. However, the dynamic nature of glacier ice makes it impractical to run a traditional photogrammetric airborne mission and generate a complete mosaic of the study site, as adjacent images acquired in a different flight line may not match due to ice movement as a result of tides and currents. A practical way to overcome this problem is to use a sampling mission, where non-overlapping images can be assumed to be representative of the study area (e.g., [33]).

We used a low-cost airborne data acquisition technique developed for photographing seals where non-overlapping simple color photos were acquired along with concurrent location information provided by the onboard global positioning system [33]. This technique does not have the fidelity of a traditional photogrammetric mission, but has the advantage of being simple, affordable, and practical. Furthermore, the objective was to quantify total ice habitat availability and fine-scale characteristics of ice in the sampled area and the precise location of each iceberg was not important.

An aerial survey of harbor seals and floating glacier ice in Johns Hopkins Inlet was conducted on 18 June 2007, during the pupping season of harbor seals. The survey was conducted from 15:19 to 16:18 hours (Alaska Daylight time), as the highest proportion of harbor seals typically haul out of the water from midday through the afternoon [29]. The survey was conducted using a de Havilland Canada DHC-2 Beaver single-engine high-winged aircraft (Ward Air Inc., Juneau, Alaska), following methods developed for counting harbor seals on icebergs in tidewater glacier fjords in Alaska [33,34]. The aircraft was flown at ~308 m (± 9.5) and ~166–176 km/h along established transects (Fig 1). The transects were programmed into the aircraft’s navigation system (Chelton Flight System) which created a 3-D image of each transect that the pilot used for navigation and to assist in maintaining the position of the aircraft along each transect line. Transects were oriented perpendicular to the face of Johns Hopkins Glacier and were spaced 200 m apart. The transects encompassed an area of approximately 8.4 km^2^ or approximately 38% of the water area of Johns Hopkins Inlet (22.0 km^2^).

Non-overlapping digital photos were taken directly under the plane using a vertically aimed digital camera (Nikon D2X) with a 60 mm focal length lens (Nikon AF MICRO). The digital camera was attached to a tripod head and mounted to a plywood platform that was secured in the belly porthole of the aircraft. The digital camera captured an image every 2 seconds, using a digital timer (Nikon MC36) that was attached to the camera and operated by the observer. The firing rate and the spacing of the transects allowed for a gap between images of ~20 m end-to-end and ~80 m side-to side thus ensuring that images were separated from one another and that areas of ice (and seals) were only sampled once during a survey. Each digital photo (3216 X 2136 pixel JPG) covered approximately 80.7 m (±2.5 SD) X 121.8 m (±3.8 SD) at the surface of the water. The images (n = 879 for 18 June 2007) were standardized to a pixel size of 0.04 m X 0.04 m using bilinear interpolation. An onboard global positioning system (GPS) (Garmin 76 CSX), with an external antenna, was used to record the track line and position of the plane along the transects (latitude, longitude, altitude) at 2-second intervals.

### Image post-processing

The latitude, longitude, and altitude from the track line were written to the EXIF headers of each digital image to permanently embed the location data in the image using RoboGeo v6.3. The EXIF header data were extracted from each image using R (R Development Core Team) and imported into ArcGIS (ESRI, version 9.3). Point shape files that contained the center-point coordinates (latitude, longitude) for each digital image were created in ArcGIS. The center points were converted to meters using Easy Calculate 10 (http://www.ian-ko.com/free/EC10/EC10_main.htm) in ArcGIS. A worldfile with the center-point coordinates was used to georeference the digital images and create a raster layer in ArcGIS. The total spatial extent of each day’s survey effort was delineated by creating a polygon that was bounded by (i) the coastline of Johns Hopkins Inlet and (ii) the approximate location of the terminus of the glacier.

Using Adobe Lightroom 6 (Adobe Systems), images were initially enhanced using a +20 contrast stretch (that pronounces the changes in brightness values within the whole image), +20 vibrance (that increases the saturation of the mid-tones in the image), followed by an image sharpening (amount 40; radius 1.0, and detail 30) to further highlight the high frequency variations (sharp edges) in the image. All images were batch-processed with these settings. Images of the glacier and of land were excluded to ensure that only floating ice (icebergs and brash ice) and water were included in the classification. Corrected images were used to develop and apply the OBIA method to quantify and characterize available ice habitat.

### Object-based image analysis and classification

**Fig 2:**
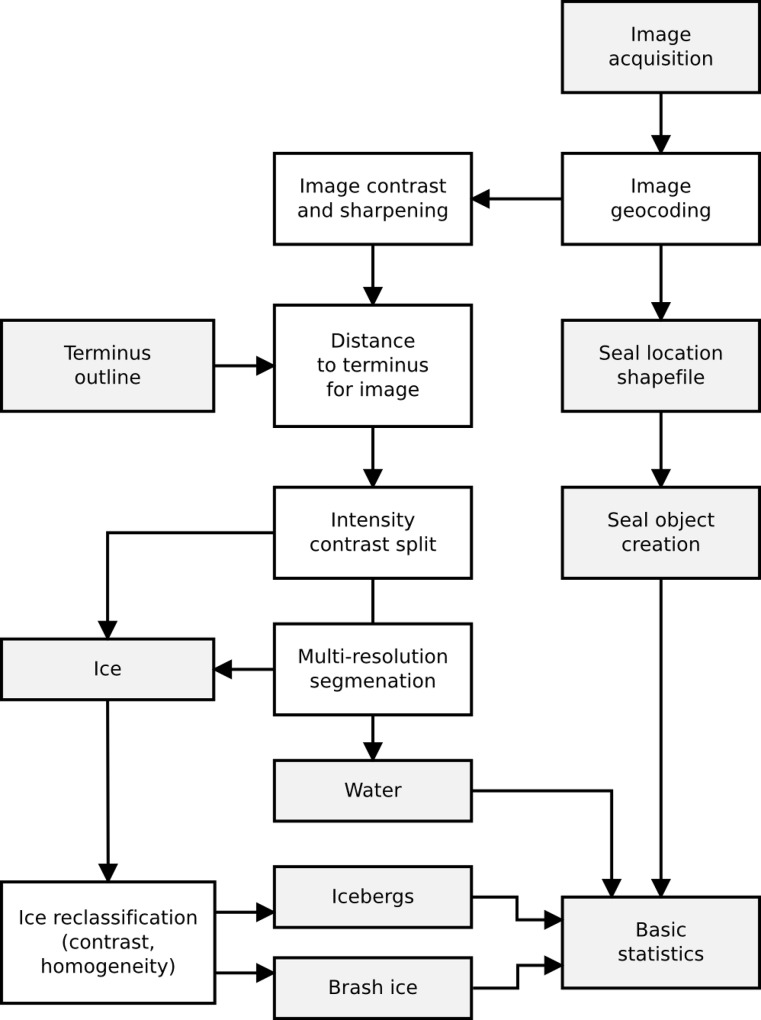
Workflow from aerial image acquisition to generation of distribution maps and statistics for seals and icebergs.

We used Trimble eCognition Developer version 8.9.0 (Trimble Geospatial Imaging) to develop and apply the OBIA workflow (Fig 2) used to classify ice, including the size and shape of individual icebergs, and water in Johns Hopkins Inlet. The variables extracted from each scene are defined in Table 1.

**Table 1:** Variables extracted from each scene using object-based image analysis.

Variable	Definition
**Iceberg (%)**	Percent of each scene that is icebergs greater than 1.6 m^2^
**Brash Ice (%)**	Percent of each scene that is ice smaller than 1.6 m^2^
**Water (%)**	Percent of each scene that is water (not ice)
**Iceberg Size (m^2^)**	Average size (m^2^) of icebergs (greater than 1.6 m^2^) in each scene
**Iceberg Angularity**	Angularity is the ratio of the perimeter of an object to the perimeter of the smallest rectangle that can enclose the object. Iceberg angularity is the average angularity for all iceberg ‘objects’ present in the scene.
**Dist. to Terminus (km)**	Distance from glacier calving face to center point of each scene

The distance between the center of each image frame and the terminus position of the glacier was calculated using existing terminus outlines from [2], when available. If necessary, additional terminus outlines were manually digitized following methods in [2] from cloud-free Landsat scenes acquired as close as possible to each survey date.

For each image, the correlation between the red, green and blue (RGB) channels was high. To reduce the redundancy of information we used a standard RGB to Intensity, Hue, Saturation (IHS) transformation. Intensity relates to scene brightness and it was more efficient to use this single channel to delineate and classify light and dark features in the scene.

Iceberg objects were initially identified by conducting a contrast split segmentation on the image intensity, which splits the image into bright and dark objects. The threshold between bright and dark objects was determined automatically by maximizing the contrast between the two classes for each image. Bright objects were then classified as icebergs, while dark objects were left unclassified at this step. To restrict our analysis to icebergs that could potentially support a seal, we filtered the iceberg objects by first merging all iceberg objects, then removing the iceberg classification from objects with fewer than 1000 pixels (corresponding to 1.6 m^2^) in size. This threshold for the minimum iceberg size was based upon the average curvilinear body length of non-pup seals (1.36 m ± 0.15 (mean ± std. dev.); range: 1.00–1.76 m; n = 81 harbor seals) that were live-captured and measured in Johns Hopkins Inlet from 2004 to 2008 [14,15], and also corresponds to estimates of minimum ice size (1 meter in diameter) used by harbor seals in Aialik Bay, a tidewater glacier fjord in southcentral Alaska [35].

**Fig 3:**
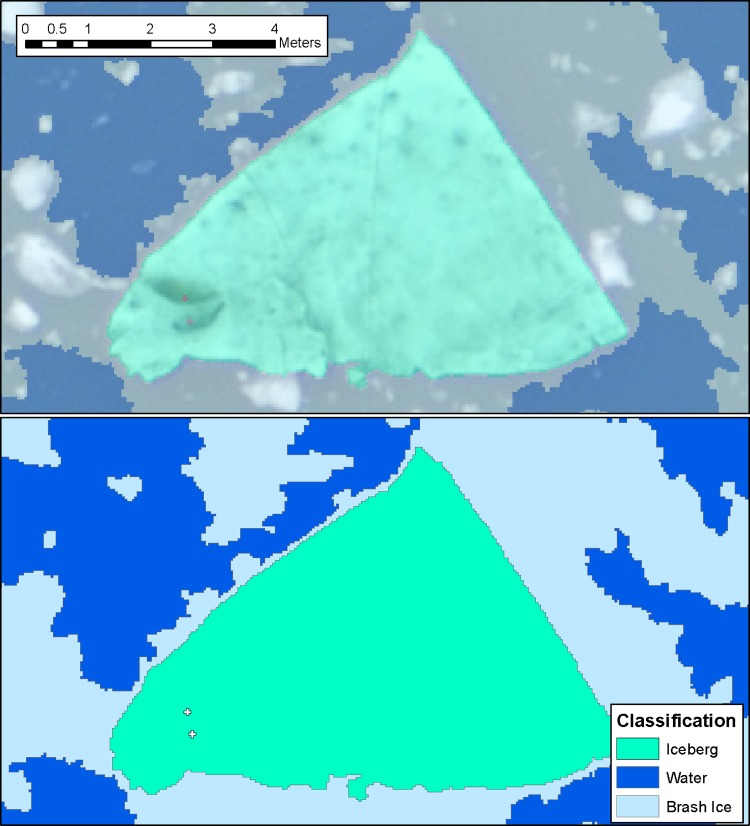
Sample classification result for the OBIA workflow. Classification, showing an iceberg, brash ice, water, and seals, is overlaid on original image. The iceberg on which the two seals are resting has an area of 27.4 m^2^ and an angularity of 1.51.

To segment the unclassified parts of the scene, we performed a multi-resolution segmentation (scale parameter: 50, shape: 0.2, compactness: 0.5). We then calculated the Grey Level Co-occurrence Matrix (GLCM) homogeneity [36] for each unclassified object, in order to distinguish between brash ice and water. Objects that were relatively smooth (GLCM homogeneity < 0.45) were classified as water. All remaining unclassified objects were classified as brash ice, here defined as any piece of ice that is less than 1.6 m^2^ in area; i.e., ice that is generally too small to support a seal. Fig 3 shows that objects classified as brash ice may contain a mixture of both ice and water pixels, depending on how densely-packed the ice is in each image.

Occasionally, the initial image segmentation resulted in the classification of “false” icebergs, or small ice pieces that were sufficiently close together to appear bright and therefore be potentially misclassified as icebergs. To address this, we performed a second contrast split segmentation on all objects classified as ice. By excluding water objects, which are typically darker, from this segmentation, we ensured that these “false” or misclassified icebergs were generally segmented into their smaller constituent pieces. To batch process the images in each survey, we used the “Analysis” function in eCognition Developer, which processes each image in the survey using eCognition Server. On average, most scenes took approximately 3–4 minutes to process. A single survey day may include up to 1,200 images, which typically takes 1–2 days to fully process; however, the time to process each survey day could be reduced by utilizing multiple licenses for eCognition Server.

**Fig 4:**
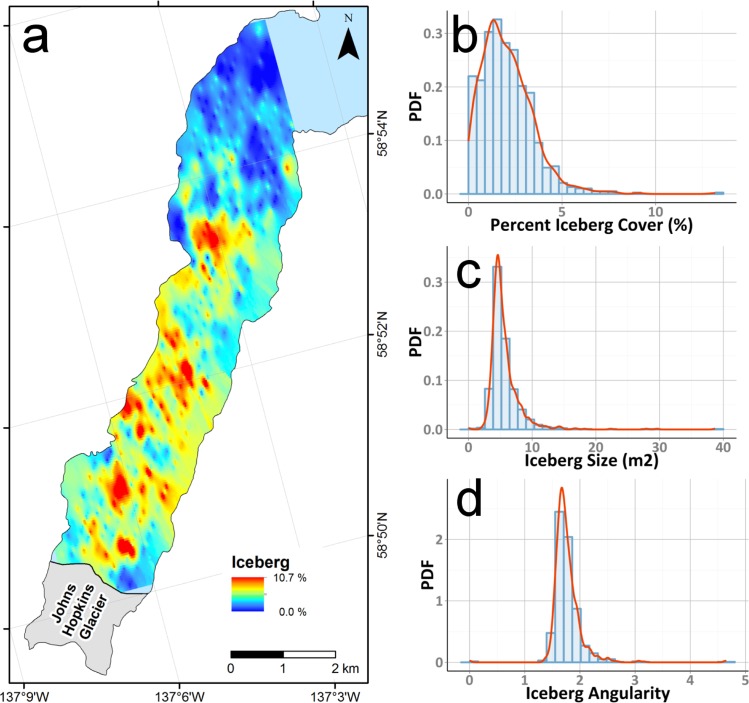
Spatial and numerical distribution of icebergs and characteristics within Johns Hopkins Inlet. (a) Distribution of icebergs (>1.6 m^2^) within Johns Hopkins Inlet on 18 June 2007, as a percentage of each aerial image. Iceberg data are interpolated from non-overlapping images. Histograms (blue) and probability density functions (red) for (b) percent iceberg cover, (c) mean iceberg size (in m^2^), and (d) mean iceberg angularity, respectively.

**Fig 5:**
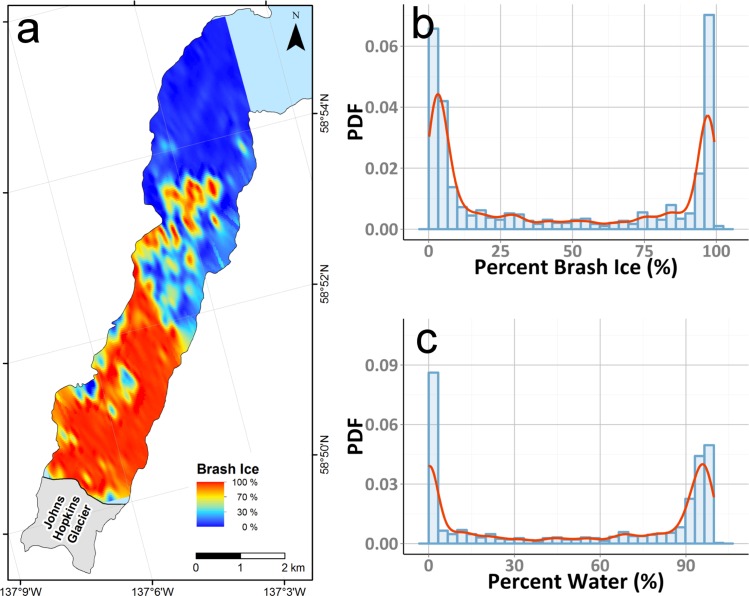
Spatial and numerical distribution of brash ice within Johns Hopkins Inlet. (a)Distribution of brash ice in Johns Hopkins Inlet on 18 June 2007, as a percentage of each aerial image. Histogram (blue) and probability density functions (PDF) (red) for percent coverage of brash ice (b) and water (c).

The classified iceberg, brash ice, and water products generated from non-overlapping images were gridded to generate a continuous interpolated surface for visualization using a Radial Basis Function (RBF). RBF is an exact operator where the interpolation surface passes through the measured points and can predict values above the maximum and below the minimum measured values. The large number of data points with slightly varying values produces the smooth surface seen in Figs 4 and 5.

### Accuracy assessment

To assess the accuracy of the OBIA classification workflow, we visually inspected and modified the classified object outlines for a random selection of 5% of the images (n = 44 images). We manually classified icebergs, brash ice, and water for each of these sample scenes by correcting the automated results. We then extracted 100 random points for each class (icebergs, brash ice, and water) from each image where that class was present, and compared the manual and automatic classifications for each point. For each class, we calculated the errors of commission (pixels incorrectly classified as a given class) and omission (pixels incorrectly not classified as a given class), as well as the producer’s and user’s accuracy [37].

## Results

We present a semi-automated method that uses OBIA to classify and quantify glacier ice floating in tidewater glacier fjords. OBIA classification of digital imagery from 18 June 2007 revealed that the sampled areas were primarily dominated by brash ice ($\bar{x}$ = 45.2%, SD = 41.5%) and water ($\bar{x}$ = 52.7%, SD = 42.3%; Table 2; Fig 4 and Fig 5). The percent brash ice was greatest near the glacier terminus and was inversely related to the distance to the glacier face (near the terminus, $\bar{x}$ = 91%, SD 16%; far from the terminus, $\bar{x}$ = 2%, SD = 3%); correspondingly, percent water increased with increasing distance from the glacier with areas near the mouth of Johns Hopkins Inlet having the highest percentage of water. On average, icebergs composed only 2.1% (SD = 1.4%) of each scene. The average iceberg size per scene was 5.7 m^2^ (SD = 2.6 m^2^); individual iceberg sizes ranged up to ~1000 m^2^. The total area of icebergs available as habitat in the sampled area was 178,700 m^2^ and for all ice (icebergs plus brash ice) was 4,078,600 m^2^. Average iceberg angularity was 1.8 (SD = 0.3) and ranged up to 4.7. An example of the classification results for a typical scene, including estimates of iceberg angularity for a sample iceberg, is provided in Fig 3. Full accuracy assessment results are summarized as a confusion matrix in Table 3. Following [37], we calculate Cohen’s Kappa statistic for these results as 0.63.

**Table 2:** Summary of results of object-based image analysis per image.

**Variables**	**Mean**	**Std. Dev.**	**Maximum**	**# of Images**
Iceberg (%)	2.1	1.4	13.2	879
Brash Ice (%)	45.2	41.5	99.3	879
Water (%)	52.7	42.3	99.8	879
Iceberg Size (m^2^)	5.7	2.6	38.7	879
Iceberg Angularity	1.8	0.3	4.7	879
Dist. to Terminus (km)	5.5	3.2	11.1	879

**Table 3:** Summary of classification error analysis results.

	Ground Truth				
	Water	Brash Ice	Icebergs	Total	User’s Accuracy
**Water**	2297	1298	0	3595	63.8%
**Brash Ice**	150	4243	4	4397	96.5%
**Icebergs**	18	1550	2831	4399	64.4%
**Total**	2465	7091	2835	12391	
**Prod. Accuracy**	93.2%	59.8%	99.9%		**Total**: 75.6%

Confusion matrix showing results of error analysis using a sample of pixels from manually-corrected classification images (“Ground Truth”) and automatically classified results.

The accuracy assessment demonstrated that the overall accuracy of this method in classifying icebergs, brash ice, and water is 75.6% (Table 3). The overall accuracy of classifying brash ice is only 59.8%, with brash ice misclassified as water or icebergs almost evenly, at 18.3% and 21.9%, respectively. This is to be expected, as brash ice objects tend to be significantly more complex than water or iceberg objects (cf. Fig 3), with some pixels representing water and others representing ice.

To estimate the accuracy of the fine-scale statistics such as iceberg size and angularity, we used a sample of the individual icebergs, from the manual classification, and compared them to iceberg objects from the automated results in the same location. The automated results underestimated total iceberg area by approximately 26% (2161 m^2^ to 2715 m^2^). Comparing values from individual icebergs from before and after manual correction, we find little to no change in the mean iceberg size (manual: $\bar{x}$ = 4.9 m^2^, SD = 7.4 m^2^; automated: $\bar{x}$ = 4.1 m^2^, SD = 8.7 m^2^) or angularity (manual: $\bar{x}$ = 1.54, SD = 0.29; automated: $\bar{x}$ = 1.57, SD = 0.35), however; suggesting that most of the area changes are due to whole icebergs being mis-classified as brash ice.

As previously stated, the total available iceberg habitat in the sampled images was 178,700 m^2^, and the total area of all ice (icebergs plus brash ice) was 4,078,600 m^2^. The uncertainty in this estimate comes primarily from misclassification of icebergs, as detailed above (26%). In addition to the classification uncertainty, there is the potential for a small bias in iceberg area due to scale distortion at the edge of each frame (< 7%, based on the flying height of the aircraft and the position of icebergs within the frame). The total uncertainty in our estimate of iceberg area that arises from these two sources is 27%, and so our final estimate of total iceberg area (± uncertainty) in the sampled images is 178,700 ± 48,200 m^2^.

There is also uncertainty associated with the assumption that our sampled images are representative of the fjord as a whole. To this end, we select 100 trials of 40 images, and calculate the mean iceberg percentage area in each sample. The resulting distribution of the mean percentage iceberg area has a mean of 2.08%, while the mean percentage iceberg area of all of the images is 2.07%, suggesting that images are well-representative of the fjord as a whole. To calculate the total area of icebergs in the fjord, then, we multiply the mean iceberg percentage (2.07%) by the area of the fjord (22 km^2^), to get a final estimate (± uncertainty) of 455,400 ± 123,000 m^2^. The standard error of the mean iceberg area is 4.66 x 10^−4^, or 10,250 m^2^ of ice.

## Conclusions

We present a semi-automated method that uses OBIA to quantify the amount and fine-scale characteristics of floating glacier ice in a tidewater glacier fjord. We have applied this method to aerial photos of Johns Hopkins Inlet in Glacier Bay National Park and Preserve, Alaska, resulting in an estimate of the total amount of icebergs in the fjord, as well as the fine-scale characteristics of individual icebergs. Understanding how the amount and characteristics of available ice may change is particularly important given that predicted changes to tidewater glacier habitats may result in changes and/or reduced availability of glacier ice habitat for wildlife, such as harbor seals [29], that depend on them as habitat.

Our accuracy analysis indicates that this method works well for classifying icebergs across scenes, with an overall classification accuracy of 75.6%. If we consider only the method performance in differentiating between ice (the two ice classes) and water, the classification method has an overall accuracy of over 88%, with producer’s and user’s accuracy for ice of 87% and 98%, respectively, suggesting that the main challenge lies in distinguishing between brash ice and icebergs. Indeed, in areas with densely-packed ice, low contrast between neighboring ice cover, or dark or sediment-covered ice, icebergs are misclassified as brash ice about 20% of the time, leading to an underestimation of total ice area by at least 20%. However, mean iceberg angularity and mean iceberg size are quite similar across our samples, further suggesting that the underestimation of total iceberg area is due to whole icebergs being classified as brash ice. In other areas where thick brash ice cover (or mélange; see, e.g., [38]) may be persistent, such as near the tidewater outlet glaciers of the Greenland Ice Sheet, further efforts may be needed to distinguish between individual pieces of ice. In areas with higher concentrations of water, the accuracy of the presented method is generally much higher. It may be possible to improve the performance by using a segmentation algorithm that focuses on shape rather than contrast to differentiate between brash ice and icebergs, as the main difference between these classes appears to be related to size, shape, and texture, rather than brightness, and this possibility will be investigated in future studies.

We provide an estimate of total iceberg area for both the sampled areas of the fjord, as well as the unsurveyed areas. Uncertainty in this estimation comes primarily from two sources: uncertainty in classification, and bias as a result of scale distortion in the aerial images. For the flying heights used to acquire the images for this study (~308 m), the uncertainty due to scale distortion is low relative to the uncertainty due to mis-classification. If this method is extended to areas where icebergs are potentially much larger (e.g., fjords in Greenland or Antarctica), or where flying heights must necessarily be much higher (due to, for example, weather conditions), these effects may be much larger; in these cases, they should be considered, and, if necessary, corrected for.

While satellite imagery has been proven to be an effective tool to count pinnipeds and assess habitat features of other pagophilic species (e.g., [39–41]), extensive cloud cover is common in our study area in southeastern Alaska and prevents the acquisition of high-quality satellite imagery on a regular basis. Very high spatial resolution aerial imagery, such as the kind used in this study, allows for the characterization of fine-scale habitat features such as estimates of size and angularity of individual icebergs. These characteristics cannot generally be determined from satellite imagery due to the limits of spatial resolution of even the best commercially available satellite images.

The OBIA technique that we have described could also be applied to imagery collected by unmanned Aerial System (UASs), which are relatively new low-cost platforms that can be used to quantify wildlife and the habitats that they use. For example, UASs have been successfully used to photograph several species and their habitats in remote regions including Steller sea lions (*Eumetopias jubatus*) in Alaska, dugongs (*Dugong dugon*) in nearshore marine habitats in Australia, and penguins and pinnipeds in Antarctic [42–44].

This study demonstrates that OBIA is a method that can be used to assess the availability of glacier ice for seals and for quantifying the fine-scale characteristics of ice in tidewater glacier fjords. An understanding of the amount and fine-scale features of icebergs will be essential for assessing how future changes in glacier calving rates may influence ice habitat that is used by harbor seals in tidewater glacier fjords. The method described here can be applied in future studies to assess seasonal and inter-annual changes in the availability of icebergs, and also used to provide quantitative estimates of iceberg characteristics that can be used in habitat and population trend models for harbor seals or other pagophilic species. The approach we have presented could be broadly applied to quantify iceberg characteristics in other tidewater glacier fjords, as well as other ice habitats such as sea ice, in order to investigate the effects of changing ice cover on other pagophilic species such as penguins and polar bears (*Ursus maritimus*).
